# Finding Cut-Offs in Leaderless Rendez-Vous Protocols is Easy

**DOI:** 10.1007/978-3-030-71995-1_3

**Published:** 2021-03-23

**Authors:** A. R. Balasubramanian, Javier Esparza, Mikhail Raskin

**Affiliations:** 8 University of Oxford, Oxford, UK; 9 Sorbonne Université - LIP6, Paris, France; Technische Universität München, Munich, Germany

**Keywords:** rendez-vous protocols, cut-off problem, Petri nets

## Abstract

In rendez-vous protocols an arbitrarily large number of indistinguishable finite-state agents interact in pairs. The cut-off problem asks if there exists a number *B* such that all initial configurations of the protocol with at least *B* agents in a given initial state can reach a final configuration with all agents in a given final state. In a recent paper [17], Horn and Sangnier prove that the cut-off problem is equivalent to the Petri net reachability problem for protocols with a leader, and in  for leaderless protocols. Further, for the special class of symmetric protocols they reduce these bounds to  and  , respectively. The problem of lowering these upper bounds or finding matching lower bounds is left open. We show that the cut-off problem is  -complete for leaderless protocols,  -complete for symmetric protocols with a leader, and in  for leaderless symmetric protocols, thereby solving all the problems left open in [17].

### 1 Introduction

Distributed systems are often designed for an unbounded number of participant agents. Therefore, they are not just one system, but an infinite family of systems, one for each number of agents. Parameterized verification addresses the problem of checking that all systems in the family satisfy a given specification.

In many application areas, agents are indistinguishable. This is the case in computational biology, where cells or molecules have no identities; in some security applications, where the agents' identities should stay private; or in applications where the identities can be abstracted away, like certain classes of multithreaded programs [15,2,31,3,18,25]. Following [3,18], we use the term *replicated systems* for distributed systems with indistinguishable agents. Replicated systems include population protocols, broadcast protocols, threshold automata, and many other models [15,2,11,7,16]. They also arise after applying a *counter abstraction* [28,3]. In finite-state replicated systems the global state of the system is determined by the function (usually called a *configuration*) that assigns

* This project has received funding from the European Research Council (ERC) under the European Union's Horizon 2020 research and innovation programme under grant agreement No 787367 (PaVeS).

to each state the number of agents that currently occupy it. This feature makes many verification problems decidable [4,10].

Surprisingly, there is no a priori relation between the complexity of a parameterized verification question (i.e., whether a given property holds for all initial configurations, or, equivalently, whether its negation holds for some configuration), and the complexity of its corresponding single-instance question (whether the property holds for a fixed initial configuration). Consider replicated systems where agents interact in pairs [15,17,2]. The complexity of single-instance questions is very robust. Indeed, checking most properties, including all properties expressible in LTL and CTL, is PSPACE-complete [9]. On the contrary, the complexity of parameterized questions is very fragile, as exemplified by the following example. While the existence of a reachable configuration that populates a given state with *at least* one agent is in P, and so well below PSPACE, the existence of a reachable configuration that populates a given state with *exactly* one agent is as hard as the reachability problem for Petri nets, and so non-elementary [6]. This fragility makes the analysis of parameterized questions very interesting, but also much harder.

Work on parameterized verification has concentrated on whether every initial configuration satisfies a given property (see e.g. [15,11,3,18,7]). However, applications often lead to questions of the form "do all initial configurations *in a given set* satisfy the property?", "do infinitely many initial configurations satisfy the property?", or "do all but finitely many initial configurations satisfy the property?". An example of the first kind is proving correctness of population protocols, where the specification requires that for a given partition $\mathcal{I}_0$, $\mathcal{I}_1$ of the set of initial configurations, and a partition $Q_0, Q_1$ of the set of states, runs starting from $\mathcal{I}_0$ eventually trap all agents within $Q_0$, and similarly for $\mathcal{I}_1$ and $Q_1$ [12]. An example of the third kind is the existence of *cut-offs*; cut-off properties state the existence of an initial configuration such that for all larger initial configurations some given property holds [8,4]. A systematic study of the complexity of these questions is still out of reach, but first results are appearing. In particular, Horn and Sangnier have recently studied the complexity of the *cut-off problem* for parameterized rendez-vous networks [17]. The problem takes as input a network with one single initial state *init* and one single final state *fin*, and asks whether there exists a cut-off $B$ such that for every number of agents $n \geq B$, the final configuration in which all agents are in state *fin* is reachable from the initial configuration in which all agents are in state *init*.

Horn and Sangnier study two versions of the cut-off problem, for leaderless networks and networks with a leader. Intuitively, a leader is a distinguished agent with its own set of states. They show that in the presence of a leader the cut-off problem and the reachability problem for Petri nets problems are inter-reducible, which shows that the cut-off problem is in the Ackermannian complexity class $\mathcal{F}_\omega$ [22], and non-elementary [6]. For the leaderless case, they show that the problem is in EXPSPACE. Further, they also consider the special case of symmetric networks, for which they obtain better upper bounds: PSPACE for the case of a

| **Horn and Sangnier** | Asymmetric rendez-vous | Symmetric rendez-vous |
|---|---|---|
| Presence of a leader | Decidable, non-elementary | PSPACE |
| Absence of a leader | EXPSPACE | NP |

| **This paper** | Asymmetric rendez-vous | Symmetric rendez-vous |
|---|---|---|
| Presence of a leader | Decidable, non-elementary | NP-complete |
| Absence of a leader | P-complete | NC |

**Table 1.** Summary of the results by Horn and Sangnier and the results of this paper.

leader, and NP in the leaderless case. These results are summarized at the top of Table 1.

In [17] the question of improving the upper bounds or finding matching lower bounds is left open. In this paper we close it with a surprising answer: All elementary upper bounds of [17] can be dramatically improved. In particular, our main result shows that the EXPSPACE bound for the leaderless case can be brought down to P. Further, the PSPACE and NP bounds of the symmetric case can be lowered to NP and NC, respectively, as shown at the bottom of Table 1. We also obtain matching lower bounds. Finally, we provide almost tight upper bounds for the size of the cut-off $B$; more precisely, we show that if $B$ exists, then $B \in 2^{n^{O(1)}}$ for a protocol of size $n$.

Our results follow from two lemmas, called the Scaling and Insertion Lemmas, that connect the *continuous semantics* for Petri nets to their standard semantics. In the continuous semantics of Petri nets transition firings can be scaled by a positive rational factor; for example, a transition can fire with factor 1/3, taking "1/3 of a token" from its input places. The continuous semantics is a relaxation of the standard one, and its associated reachability problem is much simpler (polynomial instead of non-elementary [14,6,5]). The Scaling Lemma[1] states that given two markings $M, M'$ of a Petri net, if $M'$ is reachable from $M$ in the continuous semantics, then $nM'$ is reachable from $nM$ in the standard semantics for some $n \in 2^{m^{O(1)}}$, where $m$ is the total size of the net and the markings. The Insertion Lemma states that, given four markings $M, M', L, L'$, if $M'$ is reachable from $M$ in the continuous semantics and the *marking equation* $L' = L + \mathcal{A}\mathbf{x}$ has a solution $\mathbf{x} \in \mathbb{Z}^T$ (observe that $\mathbf{x}$ can have negative components), then $nM' + L'$ is reachable from $nM + L$ in the standard semantics for some $n \in 2^{m^{O(1)}}$. We think that these lemmas can be of independent interest.

The paper is organized as follows. Section 2 contains preliminaries; in particular, it defines the cut-off problem for rendez-vous networks and reduces it to the cut-off problem for Petri nets. Section 3 gives a polynomial time algorithm for the leaderless cut-off problem for acyclic Petri nets. Section 4 introduces the Scaling and Insertion Lemmas, and Section 5 presents the novel polynomial

[1] Heavily based on previous results by Fraca and Haddad [14].

time algorithm for the cut-off problem. Sections 6 and 7 present the results for symmetric networks, for the cases with and without leaders, respectively.

Due to lack of space, full proofs of some of the lemmas can be found in the appendix.

### 2 Preliminaries

**Multisets** Let $E$ be a finite set. For a semi-ring $S$, a vector from $E$ to $S$ is a function $v : E \rightarrow S$. The set of all vectors from $E$ to $S$ will be denoted by $S^E$. In this paper, the semi-rings we will be concerned with are the natural numbers $\mathbb{N}$, the integers $\mathbb{Z}$ and the non-negative rationals $\mathbb{Q}_{\geq 0}$ (under the usual addition and multiplication operators). The *support* of a vector $v$ is the set $[\![v]\!] := \{e : v(e) \neq 0\}$ and its *size* is the number $\|v\| = \sum_{e \in [\![v]\!]} abs(v(e))$ where $abs(x)$ denotes the absolute value of $x$. Vectors from $E$ to $\mathbb{N}$ are also called discrete multisets (or just multisets) and vectors from $E$ to $\mathbb{Q}_{\geq 0}$ are called continuous multisets.

Given a multiset $M$ and a number $\alpha$ we let $\alpha \cdot M$ be the multiset given by $(\alpha \cdot M)(e) = M(e) \cdot \alpha$ for all $e \in E$. Given two multisets $M$ and $M'$ we say that $M \leq M'$ if $M(e) \leq M'(e)$ for all $e \in E$ and we let $M + M'$ be the multiset given by $(M + M')(e) = M(e) + M'(e)$ and if $M' \leq M$, we let $M - M'$ be the multiset given by $(M - M')(e) = M(e) - M'(e)$. The empty multiset is denoted by $\mathbf{0}$. We sometimes denote multisets using a set-like notation, e.g. $\wr a, 2 \cdot b, c \wr$ denotes the multiset given by $M(a) = 1, M(b) = 2, M(c) = 1$ and $M(e) = 0$ for all $e \notin \{a, b, c\}$.

Given an $I \times J$ matrix $A$ with $I$ and $J$ sets of indices, $I' \subseteq I$ and $J' \subseteq J$, we let $A_{I' \times J'}$ denote the restriction of $M$ to rows indexed by $I'$ and columns indexed by $J'$.

**Rendez-vous protocols and the cut-off problem.** Let $\Sigma$ be a fixed finite set which we will call the communication alphabet and we let $RV(\Sigma) = \{!a, ?a : a \in \Sigma\}$. The symbol $!a$ denotes that the message $a$ is sent and $?a$ denotes that the message $a$ is received.

**Definition 1.** *A rendez-vous protocol $\mathcal{P}$ is a tuple $(Q, \Sigma, init, fin, R)$ where $Q$ is a finite set of states, $\Sigma$ is the communication alphabet, $init, fin \in Q$ are the initial and final states respectively and $R \subseteq Q \times RV(\Sigma) \times Q$ is the set of rules.*

The size $|\mathcal{P}|$ of a protocol is defined as the number of bits needed to encode $\mathcal{P}$ in $\{0,1\}^*$ using some standard encoding. A configuration $C$ of $\mathcal{P}$ is a multiset of states, where $C(q)$ should be interpreted as the number of agents in state $q$. We use $\mathcal{C}(\mathcal{P})$ to denote the set of all configurations of $\mathcal{P}$. An initial (final) configuration $C$ is a configuration such that $C(q) = 0$ if $q \neq init$ (resp. $C(q) = 0$ if $q \neq fin$). We use $C^n_{init}$ ($C^n_{fin}$) to denote the initial (resp. final) configuration such that $C^n_{init}(init) = n$ (resp. $C^n_{fin}(fin) = n$).

The operational semantics of a rendez-vous protocol $\mathcal{P}$ is given by means of a transition system between the configurations of $\mathcal{P}$. We say that there is a transition between $C$ and $C'$, denoted by $C \Rightarrow C'$ iff there exists $a \in \Sigma$, $p, q, p', q' \in Q$ such that $(p, !a, p'), (q, ?a, q') \in R$, $C \geq \wr p, q \wr$ and $C' = C - \wr p, q \wr + \wr p', q' \wr$. As usual, $\xRightarrow{*}$ denotes the reflexive and transitive closure of $\Rightarrow$.

The cut-off problem for rendez-vous protocols, as defined in [17], is:

*Given:* A rendez-vous protocol $\mathcal{P}$
*Decide:* Is there $B \in \mathbb{N}$ such that $C^n_{init} \xRightarrow{*} C^n_{fin}$ for every $n \geq B$ ?

If such a $B$ exists then we say that $\mathcal{P}$ admits a cut-off and that $B$ is a cut-off for $\mathcal{P}$.

**Petri nets.** Rendez-vous protocols can be seen as a special class of Petri nets.

**Definition 2.** *A Petri net is a tuple $\mathcal{N} = (P, T, Pre, Post)$ where $P$ is a finite set of places, $T$ is a finite set of transitions, Pre and Post are matrices whose rows and columns are indexed by $P$ and $T$ respectively and whose entries belong to $\mathbb{N}$. The incidence matrix $\mathcal{A}$ of $\mathcal{N}$ is defined to be the $P \times T$ matrix given by $\mathcal{A} = Post - Pre$. Further by the* weight *of $\mathcal{N}$, we mean the largest absolute value appearing in the matrices Pre and Post.*

The size $|\mathcal{N}|$ of $\mathcal{N}$ is defined as the number of bits needed to encode $\mathcal{N}$ in $\{0, 1\}^*$ using some suitable encoding. For a transition $t \in T$ we let ${}^\bullet t = \{p : Pre[p, t] > 0\}$ and $t^\bullet = \{p : Post[p, t] > 0\}$. We extend this notation to set of transitions in the obvious way. Given a Petri net $\mathcal{N}$, we can associate with it a graph where the vertices are $P \cup T$ and the edges are $\{(p, t) : p \in {}^\bullet t\} \cup \{(t, p) : p \in t^\bullet\}$. A Petri net $\mathcal{N}$ is called acyclic if its associated graph is acyclic.

A *marking* of a Petri net is a multiset $M \in \mathbb{N}^P$, which intuitively denotes the number of *tokens* that are present in every place of the net. For $t \in T$ and markings $M$ and $M'$, we say that $M'$ is reached from $M$ by firing $t$, denoted $M \xrightarrow{t} M'$, if for every place $p$, $M(p) \geq Pre[p, t]$ and $M'(p) = M(p) + \mathcal{A}[p, t]$.

A *firing sequence* is any sequence of transitions $\sigma = t_1, t_2, \ldots, t_k \in T^*$. The support of $\sigma$, denoted by $[\![\sigma]\!]$, is the set of all transitions which appear in $\sigma$. We let $\sigma\sigma'$ denote the concatenation of two sequences $\sigma, \sigma'$.

Given a firing sequence $\sigma = t_1, t_2, \ldots, t_k \in T^*$, we let $M \xrightarrow{\sigma} M'$ denote that there exist $M_1, \ldots, M_{k-1}$ such that $M \xrightarrow{t_1} M_1 \xrightarrow{t_2} M_2 \ldots M_{k-1} \xrightarrow{t_k} M'$. Further, $M \rightarrow M'$ denotes that there exists $t \in T$ such that $M \xrightarrow{t} M'$, and $M \xrightarrow{*} M'$ denotes that there exists $\sigma \in T^*$ such that $M \xrightarrow{\sigma} M'$.

*Marking equation of a Petri net system.* In the following, a *Petri net system* is a triple $(\mathcal{N}, M, M')$ where $\mathcal{N}$ is a Petri net and $M \neq M'$ are markings. The *marking equation* for $(\mathcal{N}, M, M')$ is the equation

$$M' = M + \mathcal{A}\mathbf{v}$$

over the variables $\mathbf{v}$. It is well known that $M \xrightarrow{\sigma} M'$ implies $M' = M + \mathcal{A}\overrightarrow{\sigma}$, where $\overrightarrow{\sigma} \in \mathbb{N}^T$ is the the *Parikh image* of $\sigma$, defined as the vector whose component $\overrightarrow{\sigma}[t]$ for transition $t$ is equal to the number of times $t$ appears in $\sigma$. Therefore, if $M \xrightarrow{\sigma} M'$ then $\overrightarrow{\sigma}$ is a nonnegative integer solution of the marking equation. The converse does not hold.

**From rendez-vous protocols to Petri nets.** Let $\mathcal{P} = (Q, \Sigma, init, fin, R)$ be a rendez-vous protocol. Create a Petri net $\mathcal{N}_\mathcal{P} = (P, T, Pre, Post)$ as follows. The set of places is $Q$. For each letter $a \in \Sigma$ and for each pair of rules $r = (q, !a, s), r' = (q', ?a, s') \in R$, add a transition $t_{r,r'}$ to $\mathcal{N}_\mathcal{P}$ and set

- $Pre[p, t] = 0$ for every $p \notin \{q, q'\}$, $Post[p, t] = 0$ for every $p \notin \{s, s'\}$
- If $q = q'$ then $Pre[q, t] = -2$, otherwise $Pre[q, t] = Pre[q', t] = -1$
- If $s = s'$ then $Post[s, t] = 2$, otherwise $Post[s, t] = Post[s', t] = 1$.

It is clear that any configuration of a protocol $\mathcal{P}$ is also a marking of $\mathcal{N}_\mathcal{P}$, and vice versa. Further, the following proposition is obvious.

**Proposition 1.** *For any two configurations $C$ and $C'$ we have that $C \xRightarrow{*} C'$ over the protocol $\mathcal{P}$ iff $C \xrightarrow{*} C'$ over the Petri net $\mathcal{N}_\mathcal{P}$.*

Consequently, the cut-off problem for Petri nets, defined by

*Given :* A Petri net system $(\mathcal{N}, M, M')$
*Decide:* Is there $B \in \mathbb{N}$ such that $n \cdot M \xrightarrow{*} n \cdot M'$ for every $n \geq B$ ?

generalizes the problem for rendez-vous protocols.

### 3 The cut-off problem for acyclic Petri nets

We show that the cut-off problem for acyclic Petri nets can be solved in polynomial time. The reason for considering this special case first is that it illustrates one of the main ideas of the general case in a very pure form.

Let us fix a Petri net system $(\mathcal{N}, M, M')$ for the rest of this section, where $\mathcal{N} = (P, T, Pre, Post)$ is acyclic and $\mathcal{A}$ is its incidence matrix. It is well-known that in acyclic Petri nets the reachability relation is characterized by the marking equation (see e.g. [24]):

**Proposition 2 ([24]).** *Let $(\mathcal{N}, M, M')$ be an acyclic Petri net system. For every sequence $\sigma \in T^*$, we have $M \xrightarrow{\sigma} M'$ iff $\overrightarrow{\sigma}$ is a solution of the marking equation. Consequently, $M \xrightarrow{*} M'$ iff the marking equation has a nonnegative integer solution.*

This proposition shows that the reachability problem for acyclic Petri nets reduces to the feasibilty problem (i.e., existence of solutions) of systems of linear diophantine equations over the nonnegative integers. So the reachability problem for acyclic Petri nets is in NP, and in fact both the reachability and the feasibility problems are NP-complete [13].

There are two ways to relax the conditions on the solution so as to make the feasibility problem polynomial. Feasibility over the nonnegative *rationals* and feasibility over all integers are both in P. The first is due to the polynomiality of linear programming. For the second, feasibility can be decided in polynomial time after computing the Smith or Hermite normal forms (see e.g. [29]), which can themselves be computed in polynomial time [19]. We show that the cut-off problem can be reduced to these two relaxed problems.

#### 3.1 Characterizing acyclic systems with cut-offs

Horn and Sangnier proved in [17] a very useful charaterization of the rendez-vous protocols with a cut-off: A rendez-vous protocol $\mathcal{P}$ admits a cut-off iff there exists $n \in \mathbb{N}$ such that $C^n_{init} \stackrel{*}{\Rightarrow} C^n_{fin}$ and $C^{n+1}_{init} \stackrel{*}{\Rightarrow} C^{n+1}_{fin}$. The proof immediately generalizes to the case of Petri nets:

**Lemma 1 ([17]).** *A Petri net system $(\mathcal{N}, M, M')$ (acyclic or not) admits a cut-off iff there exists $n \in \mathbb{N}$ such that $n \cdot M \xrightarrow{*} n \cdot M'$ and $(n+1) \cdot M \xrightarrow{*} (n+1) \cdot M'$. Moreover if $n \cdot M \xrightarrow{*} n \cdot M'$ and $(n+1) \cdot M \xrightarrow{*} (n+1) \cdot M'$, then $n^2$ is a cut-off for the system.*

Using this lemma, we characterize those acyclic Petri net systems which admit a cut-off.

**Theorem 1.** *An acyclic Petri net system $(\mathcal{N}, M, M')$ admits a cut-off iff the marking equation has solutions $\mathbf{x} \in \mathbb{Q}^T_{\geq 0}$ and $\mathbf{y} \in \mathbb{Z}^T$ such that $[\![\mathbf{y}]\!] \subseteq [\![\mathbf{x}]\!]$.*

*Proof.* ($\Rightarrow$): Suppose $(\mathcal{N}, M, M')$ admits a cut-off. Hence there exists $b \in \mathbb{N}$ such that for all $n \geq b$ we have $nM \xrightarrow{*} nM'$. Let $bM \xrightarrow{\sigma'} bM'$ and $(b+1)M \xrightarrow{\tau'} (b+1)M'$. Then, notice that $(2b+1)M \xrightarrow{\sigma'\tau'} (2b+1)M'$ and $(2b+2)M \xrightarrow{\tau'\tau'} (2b+2)M'$. Hence, if we let $n = 2b+1$, $\sigma = \sigma'\tau'$ and $\tau = \tau'\tau'$ we have, $nM \xrightarrow{\sigma} nM'$, $(n+1)M \xrightarrow{\tau} (n+1)M'$ and $[\![\tau]\!] \subseteq [\![\sigma]\!]$. By Proposition 2, there exist $\mathbf{x}', \mathbf{y}' \in \mathbb{N}^T$ such that $[\![\mathbf{y}']\!] \subseteq [\![\mathbf{x}']\!]$, $nM' = nM + \mathcal{A}\mathbf{x}'$ and $(n+1)M' = (n+1)M + \mathcal{A}\mathbf{y}'$. Letting $\mathbf{x} = \mathbf{x}'/n$ and $\mathbf{y} = \mathbf{y}' - \mathbf{x}'$, we get our required vectors.

($\Leftarrow$): Suppose $\mathbf{x} \in \mathbb{Q}^T_{\geq 0}$ and $\mathbf{y} \in \mathbb{Z}^T$ are solutions of the marking equation such that $[\![\mathbf{y}]\!] \subseteq [\![\mathbf{x}]\!]$. Let $\mu$ be the least common multiple of the denominators of the components of $\mathbf{x}$, and let $\alpha$ be the largest absolute value of the numbers in the vector $\mathbf{y}$. By definition of $\mu$ we have $\alpha(\mu\mathbf{x}) \in \mathbb{N}^T$. Also, since $[\![\mathbf{y}]\!] \subseteq [\![\mathbf{x}]\!]$ it follows by definition of $\alpha$ that $\alpha(\mu\mathbf{x}) + \mathbf{y} \geq \mathbf{0}$ and hence $\alpha(\mu\mathbf{x}) + \mathbf{y} \in \mathbb{N}^T$. Since $M' = M + \mathcal{A}\mathbf{x}$ and $M' = M + \mathcal{A}\mathbf{y}$ we get

$$\alpha\mu M' = \alpha\mu M + \mathcal{A}(\alpha\mu\mathbf{x}) \qquad \text{and} \qquad (\alpha\mu+1)M' = (\alpha\mu+1)M + \mathcal{A}(\alpha\mu\mathbf{x} + \mathbf{y})$$

Taking $\alpha\mu = n$, by Proposition 2 we get that $nM \xrightarrow{*} nM'$ and $(n+1)M \xrightarrow{*} (n+1)M'$. By Lemma 1, $(\mathcal{N}, M, M')$ admits a cut-off.

Intuitively, the existence of the rational solution $\mathbf{x} \in \mathbb{Q}^T_{\geq 0}$ guarantees $nM \xrightarrow{*} nM'$ for infinitely many $n$, and the existence of the integer solution $\mathbf{y} \in \mathbb{Z}^T$ guarantees that for one of those $n$ we have $(n+1)M \xrightarrow{*} (n+1)M'$ as well.

*Example 1.* The net system given by the net on Figure 1 along with the markings $M = ⦃i⦄$ and $M' = ⦃f⦄$ admits a cut-off. The conditions of the theorem are satisfied by $\mathbf{x} = (\frac{1}{5}, \frac{1}{5}, \frac{1}{5}, \frac{1}{5})$ and $\mathbf{y} = (-1, 1, 1, 1)$.

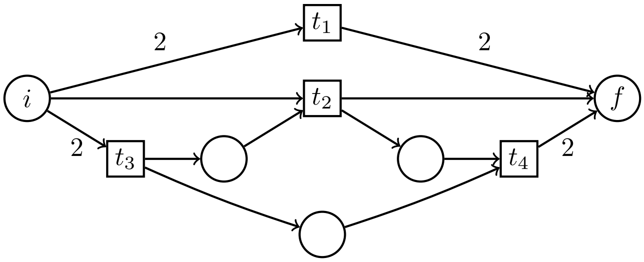

**Fig. 1.** A net with cut-off 2.

#### 3.2 Polynomial time algorithm

We derive a polynomial time algorithm for the cut-off problem from the characterization of Theorem 1. The first step is the following lemma. A very similar lemma is proved in [14], but since the proof is short we give it for the sake of completeness:

**Lemma 2.** *If the marking equation is feasible over $\mathbb{Q}_{\geq 0}$, then it has a solution with maximum support. Moreover, such a solution can be found in polynomial time.*

*Proof.* If $\mathbf{y}, \mathbf{z} \in \mathbb{Q}_{\geq 0}^T$ are solutions of the marking equation, then we have $M' = M + \mathcal{A}((\mathbf{y} + \mathbf{z})/2)$ and $[\![\mathbf{y}]\!] \cup [\![\mathbf{z}]\!] \subseteq [\![(\mathbf{y} + \mathbf{z})/2]\!]$. Hence if the marking equation if feasible over $\mathbb{Q}_{\geq 0}$, then it has a solution with maximum support.

To find such a solution in polynomial time we proceed as follows. For every transition $t$ we solve the linear program $M' = M + \mathcal{A}\mathbf{v}, \mathbf{v} \geq \mathbf{0}, \mathbf{v}(t) > 0$. (Recall that solving linear programs over the rationals can be done in polynomial time). Let $\{t_1, \ldots, t_n\}$ be the set of transitions whose associated linear programs are feasible over $\mathbb{Q}_{\geq 0}^T$, and let $\{\mathbf{u}_1, \ldots, \mathbf{u}_n\}$ be solutions to these programs. Then $1/n \cdot \sum_{i=1}^{n} \mathbf{u}_i$ is a solution of the marking equation with maximum support.

We now have all the ingredients to give a polynomial time algorithm.

**Theorem 2.** *The cut-off problem for acyclic net systems can be solved in polynomial time.*

*Proof.* First, we check that the marking equation has a solution over the non-negative rationals. If such a solution does not exist, by Theorem 1 the given net system does not admit a cut-off.

Suppose such a solution exists. By Lemma 2 we can find a non-negative rational solution $\mathbf{x}$ with maximum support in polynomial time. Let $U$ contain all the transitions $t$ such that $\mathbf{x}_t = 0$. We now check in polynomial time if the marking equation has a solution $\mathbf{y}$ over $\mathbb{Z}^T$ such that $\mathbf{y}_t = 0$ for every $t \in U$. By Theorem 1 such a solution exists iff the net system admits a cut-off.

The rendez-vous protocol given in Figure 2, which was stated in [17], is an example of a protocol where the smallest cut-off is exponential in the size of the protocol. In the next sections, we will actually prove that if a net system $\mathcal{N}$ (acyclic or not) admits a cut-off, then there is one with a polynomial number of bits in $|\mathcal{N}|$.

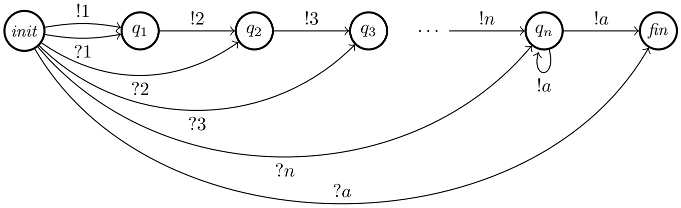

**Fig. 2.** Example of a protocol with an exponential cut-off

## 4 The Scaling and Insertion lemmas

Similar to the case of acyclic net systems, we would like to provide a characterization of net systems admitting a cut-off and then use this characterization to derive a polynomial time algorithm. Unfortunately, in general net systems there is no characterization of reachability akin to Proposition 2 for acyclic systems. To this end, we prove two intermediate lemmas to help us come up with a characterization for cut-off admissible net systems in the general case. We believe that these two lemmas could be of independent interest in their own right. Further, the proofs of both lemmas are provided so that it will enable us later on to derive a bound on the cut-off for net systems.

### 4.1 The Scaling Lemma

The Scaling Lemma shows that, given a Petri net system $(\mathcal{N}, M, M')$, whether $nM \xrightarrow{*} nM'$ holds for some $n \geq 1$ can be decided in polynomial time; moreover, if $nM \xrightarrow{*} nM'$ holds for some $n$, then it holds for some $n$ with at most $(|\mathcal{N}|(\log \|M\| + \log \|M'\|))^{O(1)}$ bits. The name of the lemma is due to the fact that the firing sequence leading from $nM$ to $nM'$ is obtained by *scaling up* a *continuous firing sequence* from $M$ to $M'$; the existence of such a continuous sequence can be decided in polynomial time [14].

In the rest of the section we first recall continuous Petri nets and the charaterization of [14], and then present the Scaling Lemma[2].

[2] The lemma is implicitly proved in [14], but the bound on the size of $n$ is hidden in the details of the proof, and we make it explicit.

**Reachability in continuous Petri nets.** Petri nets can be given a *continuous semantics* (see e.g. [1,30,14]), in which markings are continuous multisets; we call them *continuous markings*. A continuous marking $M$ enables a transition $t$ *with factor* $\lambda \in \mathbb{Q}_{\geq 0}$ if $M(p) \geq \lambda \cdot Pre[p,t]$ for every place $p$; we also say that $M$ enables $\lambda t$. If $M$ enables $\lambda t$, then $\lambda t$ can fire or occur, leading to a new marking $M'$ given by $M'(p) = M(p) + \lambda \cdot \mathcal{A}[p,t]$ for every $p \in P$. We denote this by $M \xrightarrow[\mathbb{Q}]{\lambda t} M'$, and say that $M'$ is reached from $M$ by firing $\lambda t$. A *continuous firing sequence* is any sequence of transitions $\sigma = \lambda_1 t_1, \lambda_2 t_2, \ldots, \lambda_k t_k \in (\mathbb{Q}_{\geq 0} \times T)^*$. We let $M \xrightarrow[\mathbb{Q}]{\sigma} M'$ denote that there exist continuous markings $M_1, \ldots, M_{k-1}$ such that $M \xrightarrow[\mathbb{Q}]{\lambda_1 t_1} M_1 \xrightarrow[\mathbb{Q}]{\lambda_2 t_2} M_2 \cdots M_{k-1} \xrightarrow[\mathbb{Q}]{\lambda_k t_k} M'$. Further, $M \xrightarrow[\mathbb{Q}]{*} M'$ denotes that $M \xrightarrow[\mathbb{Q}]{\sigma} M'$ holds for some continuous firing sequence $\sigma$.

The *Parikh image* of $\sigma = \lambda_1 t_1, \lambda_2 t_2, \ldots, \lambda_k t_k \in (\mathbb{Q}_{\geq 0} \times T)^*$ is the vector $\overrightarrow{\sigma} \in \mathbb{Q}^T_{\geq 0}$ where $\overrightarrow{\sigma}[t] = \sum_{i=1}^k \delta_{i,t} \lambda_i$, where $\delta_{i,t} = 1$ if $t_i = t$ and 0 otherwise. The support of $\sigma$ is the support of its Parikh image $\overrightarrow{\sigma}$. If $M \xrightarrow[\mathbb{Q}]{\sigma} M'$ then $\overrightarrow{\sigma}$ is a solution of the marking equation over $\mathbb{Q}^T_{\geq 0}$, but the converse does not hold. In [14], Fraca and Haddad strengthen this necessary condition to make it also sufficient, and use the resulting characterization to derive a polynomial algorithm.

**Theorem 3 ([14]).** *Let $(\mathcal{N}, M, M')$ be a Petri net system.*

- *$M \xrightarrow[\mathbb{Q}]{\sigma} M'$ iff $\overrightarrow{\sigma}$ is a solution of the marking equation over $\mathbb{Q}^T_{\geq 0}$, and there exist continuous firing sequences $\tau$, $\tau'$ and continuous markings $L$ and $L'$ such that $[\![\tau]\!] = [\![\sigma]\!] = [\![\tau']\!]$, $M \xrightarrow[\mathbb{Q}]{\tau} L$, and $L' \xrightarrow[\mathbb{Q}]{\tau'} M'$.*
- *It can be decided in polynomial time if $M \xrightarrow[\mathbb{Q}]{*} M'$ holds.*

**Scaling.** It follows easily from the definitions that $nM \xrightarrow{*} nM'$ holds for some $n \geq 1$ iff $M \xrightarrow[\mathbb{Q}]{*} M'$. Indeed, if $M \xrightarrow[\mathbb{Q}]{\sigma} M'$ for some $\sigma = \lambda_1 t_1, \lambda_2 t_2, \ldots, \lambda_k t_k \in (\mathbb{Q}_{\geq 0} \times T)^*$, then we can scale this continuous firing sequence to a discrete sequence $nM \xrightarrow[\mathbb{Q}]{n\sigma} nM'$ where $n$ is the smallest number such that $n\lambda_1, \ldots, n\lambda_k \in \mathbb{N}$, and $n\sigma = t_1^{n\lambda_1} t_2^{n\lambda_2} \ldots t_k^{n\lambda_k}$. So Theorem 3 immediately implies that the existence of $n \geq 1$ such that $nM \xrightarrow{*} nM'$ can be decided in polynomial time. The following lemma also gives a bound on $n$.

**Lemma 3.** *Let $(\mathcal{N}, M, M')$ be a Petri net system with weight $w$ such that $M \xrightarrow[\mathbb{Q}]{\sigma} M'$ for some continuous firing sequence $\sigma \in (\mathbb{Q}_{\geq 0} \times T)^*$. Let $m$ be the number of transitions in $[\![\sigma]\!]$ and let $\ell$ be $\|\overrightarrow{\sigma}\|$. Let $k$ be the smallest natural number such that $k\overrightarrow{\sigma} \in \mathbb{N}^T$. Then, there exists a firing sequence $\tau \in T^*$ such that $[\![\tau]\!] = [\![\sigma]\!]$ and*

$$\left(16w(w+1)^{2m}k\ell \cdot M\right) \xrightarrow{\tau} \left(16w(w+1)^{2m}k\ell \cdot M'\right)$$

**Lemma 4. (Scaling Lemma).** *Let $(\mathcal{N}, M, M')$ be a Petri net system such that $M \xrightarrow[\mathbb{Q}]{\sigma} M'$. There exists a number $n$ with a polynomial number of bits in $|\mathcal{N}|(\log \|M\| + \log \|M'\|)$ such that $nM \xrightarrow{\tau} nM'$ for some $\tau$ with $[\![\tau]\!] = [\![\sigma]\!]$.*

#### 4.2 The Insertion Lemma

In the acyclic case, the existence of a cut-off is characterized by the existence of solutions to the marking equation $\mathbb{Q}^T_{\geq 0}$ and $\mathbb{Z}^T$. Intuitively, in the general case we replace the existence of solutions over $\mathbb{Q}^T_{\geq 0}$ by the conditions of the Scaling Lemma, and the existence of solutions over $\mathbb{Z}^T$ by the Insertion Lemma:

**Lemma 5 (Insertion Lemma).** *Let $M, M', L, L'$ be markings of $\mathcal{N}$ satisfying $M \xrightarrow{\sigma} M'$ for some $\sigma \in T^*$ and $L' = L + \mathcal{A}\mathbf{y}$ for some $\mathbf{y} \in \mathbb{Z}^T$ such that $[\![\mathbf{y}]\!] \subseteq [\![\sigma]\!]$. Then $\mu M + L \xrightarrow{*} \mu M' + L'$ for $\mu = \|\mathbf{y}\|(\|\overrightarrow{\sigma}\|nw + nw + 1)$ , where $w$ is the weight of $\mathcal{N}$, and $n$ is the number of places in ${}^\bullet[\![\sigma]\!]$.*

The idea of the proof is a follows: In a first stage, we asynchronously execute multiple "copies" of the firing sequence $\sigma$ from multiple "copies" of the marking $M$, until we reach a marking at which all places of ${}^\bullet[\![\sigma]\!]$ contain a sufficiently large number of tokens. At this point we temporarily interrupt the executions of the copies of $\sigma$ to *insert* a firing sequence with Parikh mapping $\|\mathbf{y}\|\overrightarrow{\sigma} + \mathbf{y}$. The net effect of this sequence is to transfer some copies of $M$ to $M'$, leaving the other copies untouched, and exactly one copy of $L$ to $L'$. In the third stage, we resume the interrupted executions of the copies of $\sigma$, which completes the transfer of the remaining copies of $M$ to $M'$ .

*Proof.* Let $\mathbf{x}$ be the Parikh image of $\sigma$, i.e., $\mathbf{x} = \overrightarrow{\sigma}$. Since $M \xrightarrow{\sigma} M'$, by the marking equation we have $M' = M + \mathcal{A}\mathbf{x}$

**First stage:** Let $\lambda_x = \|x\|$, $\lambda_y = \|y\|$ and $\mu = \lambda_y(\lambda_x nw + nw + 1)$. Let $\sigma := r_1, r_2, \ldots, r_k$ and let $M =: M_0 \xrightarrow{r_1} M_1 \xrightarrow{r_2} M_2 \ldots M_{k-1} \xrightarrow{r_k} M_k := M$. Notice that for each place $p \in {}^\bullet[\![\sigma]\!]$, there exists a marking $M_{i_p} \in \{M_0, \ldots, M_{k-1}\}$ such that $M_{i_p}(p) > 0$.

Since each of the markings in $\{M_{i_p}\}_{p \in {}^\bullet[\![\sigma]\!]}$ can be obtained from $M$ by firing a (suitable) prefix of $\sigma$, it is easy to see that from the marking $\mu M + L = \lambda_y M + L + (\lambda_x \lambda_y nw + \lambda_y nw)M$ we can reach the marking $\texttt{First} := \lambda_y M + L + \sum_{p \in {}^\bullet[\![\sigma]\!]} (\lambda_x \lambda_y w + \lambda_y w) M_{i_p}$. This completes our first stage.

**Second stage - Insert:** Since $[\![\mathbf{y}]\!] \subseteq [\![\sigma]\!]$, if $\mathbf{y}(t) \neq 0$ then $\mathbf{x}(t) \neq 0$. Since $\mathbf{x}(t) \geq 0$ for every transition, it now follows that $(\lambda_y \mathbf{x} + \mathbf{y})(t) \geq 0$ for every transition $t$ and $(\lambda_y \mathbf{x} + \mathbf{y})(t) > 0$ precisely for those transitions in $[\![\sigma]\!]$.

Let $\xi$ be any firing sequence such that $\overrightarrow{\xi} = \lambda_y \mathbf{x} + \mathbf{y}$. Notice that for every place $p \in {}^\bullet[\![\sigma]\!]$, $\texttt{First}(p) \geq \lambda_x \lambda_y w + \lambda_y w \geq \|(\lambda_y \mathbf{x} + \mathbf{y})\| \cdot w$. By an easy induction on $\|\xi\|$, it follows that that $\texttt{First} \xrightarrow{\xi} \texttt{Second}$ for some marking $\texttt{Second}$. By the marking equation, it follows that $\texttt{Second} = \lambda_y M' + L' + \sum_{p \in {}^\bullet[\![\sigma]\!]} (\lambda_x \lambda_y w + \lambda_y w) M_{i_p}$. This completes our second stage.

**Third stage:** Notice that for each place $p \in {}^\bullet[\![\sigma]\!]$, by construction of $M_{i_p}$, there is a firing sequence which takes the marking $M_{i_p}$ to the marking $M'$. It then follows that there is a firing sequence which takes the marking $\texttt{Second}$ to the marking $\lambda_y M' + L' + \sum_{p \in {}^\bullet[\![\sigma]\!]} (\lambda_x \lambda_y w + \lambda_y w) M' = \mu M' + L'$. This completes our third stage and also completes the desired firing sequence from $\mu M + L$ to $\mu M' + L'$.

## 5 Polynomial time algorithm for the general case

Let $(\mathcal{N}, M, M')$ be a net system with $\mathcal{N} = (P, T, Pre, Post)$, such that $\mathcal{A}$ is its incidence matrix. As in Section 3, we first characterize the Petri net systems that admit a cut-off, and then provide a polynomial time algorithm.

### 5.1 Characterizing systems with cut-offs

We generalize the characterization of Theorem 1 for acyclic Petri net systems to general systems.

**Theorem 4.** *A Petri net system $(\mathcal{N}, M, M')$ admits a cut-off iff there exists some rational firing sequence $\sigma$ such that $M \xrightarrow[\mathbb{Q}]{\sigma} M'$ and the marking equation has a solution $\mathbf{y} \in \mathbb{Z}^T$ such that $[\![\mathbf{y}]\!] \subseteq [\![\sigma]\!]$.*

*Proof.* ($\Rightarrow$): Assume $(\mathcal{N}, M, M')$ admits a cut-off. Hence there exists $B \in \mathbb{N}$ such that for all $n \geq B$ we have $nM \xrightarrow{*} nM'$. Similar to the proof of theorem 1, we can show that there exist $n \in \mathbb{N}$ and firing sequences $\tau, \tau'$ such that $nM \xrightarrow{\tau} nM'$, $(n+1)M \xrightarrow{\tau'} (n+1)M'$ and $[\![\tau']\!] \subseteq [\![\tau]\!]$.

Let $\tau = t_1 t_2 \cdots t_k$. Construct the rational firing sequence $\sigma := t_1/n\, t_2/n \cdots t_k/n$. From the fact that $nM \xrightarrow{\tau} nM'$, we can easily conclude by induction on $k$ that $M \xrightarrow[\mathbb{Q}]{\sigma} M'$. Further, by the marking equation we have $nM' = nM + \mathcal{A}\overrightarrow{\tau}$ and $(n+1)M' = (n+1)M + \mathcal{A}\overrightarrow{\tau'}$. Let $\mathbf{y} = \overrightarrow{\tau'} - \overrightarrow{\tau}$. Then $\mathbf{y} \in \mathbb{Z}^T$ and $M' = M + \mathcal{A}\mathbf{y}$. Further, since $[\![\tau']\!] \subseteq [\![\tau]\!] = [\![\sigma]\!]$, we have $[\![\mathbf{y}]\!] \subseteq [\![\sigma]\!]$.

($\Leftarrow$): Assume there exists a rational firing sequence $\sigma$ and a vector $\mathbf{y} \in \mathbb{Z}^T$ such that $[\![\mathbf{y}]\!] \subseteq [\![\sigma]\!]$, $M \xrightarrow[\mathbb{Q}]{\sigma} M'$ and $M' = M + \mathcal{A}\mathbf{y}$. Let $s = |\mathcal{N}|(\log\|M\| + \log\|M'\|)$. It is well known that if a system of linear equations over the integers is feasible, then there is a solution which can be described using a number of bits which is polynomial in the size of the input (see e.g. [20]). Hence, we can assume that $\|\mathbf{y}\|$ can be described using $s^{O(1)}$ bits.

By Lemma 4 there exists $n$ (which can be described using $s^{O(1)}$ bits) and a firing sequence $\tau$ with $[\![\tau]\!] = [\![\sigma]\!]$ such that $nM \xrightarrow{\tau} nM'$. Hence $knM \xrightarrow{*} knM'$ is also possible for any $k \in \mathbb{N}$. By Lemma 5, there exists $\mu$ (which can once again be described using $s^{O(1)}$ bits) such that $\mu nM + M \xrightarrow{*} \mu nM' + M'$ is possible. By Lemma 1 the system $(\mathcal{N}, M, M')$ admits a cut-off with a polynomial number of bits in $s$.

Notice that we have actually proved that if a net system admits a cut-off then it admits a cut-off with a polynomial number of bits in its size. Since the cut-off problem for a rendez-vous protocol $\mathcal{P}$ can be reduced to a cut-off problem for the Petri net system $(\mathcal{N}_\mathcal{P}, \llparenthesis init \rrparenthesis, \llparenthesis fin \rrparenthesis)$, it follows that,

**Corollary 1.** *If the system $(\mathcal{N}, M, M')$ admits a cut-off then it admits a cut-off with a polynomial number of bits in $|\mathcal{N}|(\log\|M\| + \log\|M'\|)$. Hence, if a rendez-vous protocol $\mathcal{P}$ admits a cut-off then it admits a cut-off with a polynomial number of bits in $|\mathcal{P}|$.*

#### 5.2 Polynomial time algorithm

We use the characterization given in the previous section to provide a polynomial time algorithm for the cut-off problem. The following lemma, which was proved in [14] and whose proof is given in the appendix, enables us to find a firing sequence between two markings with maximum support.

**Lemma 6.** *[14] Among all the rational firing sequences $\sigma$ such that $M \xrightarrow[\mathbb{Q}]{\sigma} M'$, there is one with maximum support. Moreover, the support of such a firing sequence can be found in polynomial time.*

We now have all the ingredients to prove the existence of a polynomial time algorithm.

**Theorem 5.** *The cut-off problem for net systems can be solved in polynomial time.*

*Proof.* First, we check that there is a rational firing sequence $\sigma$ with $M \xrightarrow[\mathbb{Q}]{\sigma} M'$, which can be done in polynomial time by ([14], Proposition 27). If such a sequence does not exist, by Theorem 4 the given net system does not admit a cut-off.

Suppose such a sequence exists. By Lemma 6 we can find in polynomial time, the maximum support $S$ of all the firing sequences $\tau$ such that $M \xrightarrow[\mathbb{Q}]{\tau} M'$. We now check in polynomial time if the marking equation has a solution $\mathbf{y}$ over $\mathbb{Z}^T$ such that $\mathbf{y}(t) = 0$ for every $t \notin S$. By Theorem 4 such a solution exists iff the net system admits a cut-off.

This immediately proves that the cut-off problem for rendez-vous protocols is also in polynomial time. By an easy logspace reduction from the Circuit Value Problem [21], we prove that

**Lemma 7.** *The cut-off problem for rendez-vous protocols is P-hard.*

Clearly, this also proves that the cut-off problem for Petri nets is P-hard.

### 6 Symmetric rendez-vous protocols

In [17] Horn and Sangnier introduce symmetric rendez-vous protocols, where sending and receiving a message at each state has the same effect, and show that the cut-off problem is in $\mathsf{NP}$. We improve on their result and shown that it is in $\mathsf{NC}$.

Recall that $\mathsf{NC}$ is the set of problems in $\mathsf{P}$ that can be solved in polylogarithmic *parallel* time, i.e., problems which can be solved by a uniform family of circuits with polylogarithmic depth and polynomial number of gates. Two well-known problems which lie in $\mathsf{NC}$ are graph reachability and feasibility of linear equations over the finite field $\mathbb{F}_2$ of size 2 [27,23]. We proceed to formally define symmetric protocols and state our results.

**Definition 3.** *A rendez-vous protocol* $\mathcal{P} = (Q, \Sigma, init, fin, R)$ *is* symmetric*, iff its set of rules is symmetric under swapping* $!a$ *and* $?a$ *for each* $a \in \Sigma$*, i.e., for each* $a \in \Sigma$*, we have* $(q, !a, q') \in R$ *iff* $(q, ?a, q') \in R$.

Horn and Sangnier show that, because of their symmetric nature, there is a very easy characterization for cut-off admitting symmetric protocols.

**Proposition 3.** *([17], Lemma 18) A symmetric protocol* $\mathcal{P}$ *admits a cut-off iff there exists an even number* $e$ *and an odd number* $o$ *such that* $C^e_{init} \xrightarrow{*} C^e_{fin}$ *and* $C^o_{init} \xrightarrow{*} C^o_{fin}$.

From a symmetric protocol $\mathcal{P}$, we can derive a graph $G(\mathcal{P})$ where the vertices are the states and there is an edge between $q$ and $q'$ iff there exists $a \in \Sigma$ such that $(q, a, q') \in R$. The following proposition is immediate from the definition of symmetric protocols:

**Proposition 4.** *Let* $\mathcal{P}$ *be a symmetric protocol. There exists an even number* $e$ *such that* $C^e_{init} \xrightarrow{*} C^e_{fin}$ *iff there is a path from init to fin in the graph* $G(\mathcal{P})$.

*Proof.* The left to right implication is obvious. For the other side, suppose there is a path $init, q_1, q_2, \dots, q_{m-1}, fin$ in the graph $G(\mathcal{P})$. Then notice that $\wr 2 \cdot init \wr \rightarrow \wr 2 \cdot q_1 \wr \rightarrow \wr 2 \cdot q_2 \wr \cdots \rightarrow \wr 2 \cdot q_{m-1} \wr \rightarrow \wr 2 \cdot q_f \wr$ is a valid run of the protocol.

Since graph reachability is in NC , this takes care of the "even" case from Proposition 3. Hence, we only need to take care of the "odd" case from Proposition 3.

Fix a symmetric protocol $\mathcal{P}$ for the rest of the section. As a first step, for each state $q \in Q$, we compute if there is a path from *init* to $q$ and if there is a path from $q$ to *fin* in the graph $\mathcal{G}(\mathcal{P})$. Since graph reachability is in NC this computation can be carried out in NC by parallely running graph reachability for each $q \in Q$. If such paths exist for a state $q$ then we call $q$ a good state, and otherwise a bad state. The following proposition easily follows from the symmetric nature of $\mathcal{P}$:

**Proposition 5.** *If* $q \in Q$ *is a good state, then* $\wr 2 \cdot init \wr \xrightarrow{*} \wr 2 \cdot q \wr$ *and* $\wr 2 \cdot q \wr \xrightarrow{*} \wr 2 \cdot fin \wr$.

Similar to the general case of rendez-vous protocols, given a symmetric protocol $\mathcal{P}$ we can construct a Petri net $\mathcal{N}_{\mathcal{P}}$ whose places are the states of $\mathcal{P}$ and which faithfully represents the reachability relation of configurations of $\mathcal{P}$. Observe that this construction can be carried out in parallel over all the states in $Q$ and over all pairs of rules in $R$. Let $\mathcal{N} = (P, T, Pre, Post)$ be the Petri net that we construct out of the symmetric protocol $\mathcal{P}$ and let $\mathcal{A}$ be its incidence matrix. We now write the marking equation for $\mathcal{N}$ as follows: We introduce a variable $\mathbf{v}[t]$ for each transition $t \in T$ and we construct an equation system $Eq$ enforcing the following three conditions:

- $\mathbf{v}[t] = 0$ for every $t \in T$ such that ${}^\bullet t \cup t^\bullet$ contains a bad state.
  By definition of a bad state, such transitions will never be fired on any run from an initial to a final configuration and so our requirement is safe.

– $\sum_{t \in T} \mathcal{A}[q,t] \cdot \mathbf{v}[t] = 0$ for each $q \notin \{init, fin\}$.
  Notice that the net-effect of any run from an initial to a final configuration on any state not in $\{init, fin\}$ is 0 and hence this condition is valid as well.
– $\sum_{t \in T} \mathcal{A}[init,t] \cdot \mathbf{v}[t] = -1$ and $\sum_{t \in T} \mathcal{A}[fin,t] \cdot \mathbf{v}[t] = 1$.

It is clear that the construction of $Eq$ can be carried out in parallel over each $q \in Q$ and each $t \in T$. Finally, we solve $Eq$ over arithmetic modulo 2, i.e., we solve $Eq$ over the field $\mathbb{F}_2$ which as mentioned before can be done in NC. We have:

**Lemma 8.** *There exists an odd number $o$ such that $C^o_{init} \xrightarrow{*} C^o_{fin}$ iff the equation system $Eq$ has a solution over $\mathbb{F}_2$.*

*Proof.* (Sketch.) The left to right implication is true because of taking modulo 2 on both sides of the marking equation. For the other side, we use an idea similar to Lemma 5. Let $\mathbf{x}$ be a solution to $Eq$ over $\mathbb{F}_2$. Using Proposition 5 we first populate all the good states of $Q$ with enough processes such that all the good states except *init* have an even number of processes. Then, we fire exactly once, all the transitions $t$ such that $\mathbf{x}[t] = 1$. Since $\mathbf{x}$ satisfies $Eq$, we can now argue that in the resulting configuration, the number of processes at each bad state is 0 and the number of processes in each good state except *fin* is even. Hence, we can once again use Proposition 5 to conclude that we can move all the processes which are not at *fin* to the final state *fin*.

**Theorem 6.** *The problem of deciding whether a symmetric protocol admits a cut-off is in* NC.

*Proof.* By Proposition 3 it suffices to find an even number $e$ and an odd number $o$ such that $C^e_{init} \xrightarrow{*} C^e_{fin}$ and $C^o_{init} \xrightarrow{*} C^o_{fin}$. By Proposition 4 the former can be done in NC. By Lemma 8 and by the fact that the equation system $Eq$ can be constructed and solved in NC, it follows that the latter can also be done in NC.

### 7 Symmetric protocols with leaders

In this section, we extend symmetric rendez-vous protocols by adding a special process called leader. We state the cut-off problem for such protocols and prove that it is NP-complete.

**Definition 4.** *A symmetric leader protocol is a pair of symmetric protocols $\mathcal{P} = (\mathcal{P}^L, \mathcal{P}^F)$ where $\mathcal{P}^L = (Q^L, \Sigma, init^L, fin^L, R^L)$ is the leader protocol and $\mathcal{P}^F = (Q^F, \Sigma, init^F, fin^F, R^F)$ is the follower protocol where $Q^L \cap Q^F = \emptyset$.*

A configuration of a symmetric leader protocol $\mathcal{P}$ is a multiset over $Q^L \cup Q^F$ such that $\sum_{q \in Q^L} C(q) = 1$. This corresponds to the intuition that exactly one process can execute the leader protocol. For each $n \in \mathbb{N}$, let $C^n_{init}$ (resp. $C^n_{fin}$) denote the initial (resp. final) configuration of $\mathcal{P}$ given by $C^n_{init}(init^L) = 1$ (resp. $C^n_{fin}(fin^L) = 1$) and $C^n_{init}(init^F) = n$ (resp. $C^n_{fin}(fin^F) = n$). We say that $C \Rightarrow C'$

if there exists $(p, !a, p'), (q, ?a, q') \in R^L \cup R^F$, $C \geq \wr p, q \wr$ and $C' = C - \wr p, q \wr + \wr p', q' \wr$. Since we allow at most one process to execute the leader protocol, given a configuration $C$, we can let $lead(C)$ denote the unique state $q \in Q^L$ such that $C(q) > 0$.

**Definition 5.** *The cut-off problem for symmetric leader protocols is the following.*

*Input: A symmetric leader protocol* $\mathcal{P} = (\mathcal{P}^L, \mathcal{P}^F)$.
*Output: Is there* $B \in \mathbb{N}$ *such that for all* $n \geq B$, $C^n_{init} \xrightarrow{*} C^n_{fin}$.

We know the following fact regarding symmetric leader protocols.

**Proposition 6.** *([17], Lemma 18) A symmetric leader protocol admits a cut-off iff there exists an even number* $e$ *and an odd number* $o$ *such that* $C^e_{init} \xrightarrow{*} C^e_{fin}$ *and* $C^o_{init} \xrightarrow{*} C^o_{fin}$.

The main theorem of this section is

**Theorem 7.** *The cut-off problem for symmetric leader protocols is* NP*-complete*

#### 7.1 A non-deterministic polynomial time algorithm

Let $\mathcal{P} = (\mathcal{P}^L, \mathcal{P}^F)$ be a symmetric leader protocol with $\mathcal{P}^L = (Q^L, \Sigma, init^L, fin^L, R^L)$ and $\mathcal{P}^F = (Q^F, \Sigma, init^F, fin^F, R^F)$. Similar to the previous section, from $\mathcal{P}^F$ we can construct a graph $\mathcal{G}(\mathcal{P}^F)$ where the vertices are given by the states $Q^F$ and the edges are given by the rules in $R^F$. In $\mathcal{G}(\mathcal{P}^F)$, we can clearly remove all vertices which are not reachable from the state $init^F$ and which do not have a path to $fin^F$. In the sequel, we will assume that such vertices do not exist in $\mathcal{G}(\mathcal{P}^F)$.

Similar to the general case, we will construct a Petri net $\mathcal{N}_\mathcal{P}$ from the given symmetric leader protocol $\mathcal{P}$. However, the construction is made slightly complicated due to the presence of a leader.

From $\mathcal{P} = (\mathcal{P}^L, \mathcal{P}^F)$, we construct a Petri net $\mathcal{N} = (P, T, Pre, Post)$ as follows: Let $P$ be $Q^L \cup Q^F$. For each $a \in \Sigma$ and $r = (q, !a, s), r' = (q', ?a, s') \in R^L \cup R^F$ such that *at most one of* $r$ *and* $r'$ *belongs to* $R^L$, we will have a transition $t_{r,r'} \in T$ in $\mathcal{N}$ such that

- $Pre[p, t] = 0$ for every $p \notin \{q, q'\}$, $Post[p, t] = 0$ for every $p \notin \{s, s'\}$
- If $q = q'$ then $Pre[q, t] = -2$, otherwise $Pre[q, t] = Pre[q', t] = -1$
- If $s = s'$ then $Post[s, t] = 2$, otherwise $Post[s, t] = Post[s', t] = 1$.

Transitions $t_{r,r'}$ in which exactly one of $r, r'$ is in $R^L$ will be called *leader transitions* and transitions in which both of $r, r'$ are in $R^F$ will be called *follower-only transitions*. Notice that if $t$ is a leader transition, then there is a unique place $p \in {}^\bullet t \cap Q^L$ and a unique place $p \in t^\bullet \cap Q^L$. These places will be denoted by $t.from$ and $t.to$ respectively.

As usual, we let $\mathcal{A}$ denote the incidence matrix of the constructed net $\mathcal{N}$. The following proposition is obvious from the construction of the net $\mathcal{N}$

**Proposition 7.** *For two configurations $C$ and $C'$, we have that $C \overset{*}{\Rightarrow} C'$ in the protocol $\mathcal{P}$ iff $C \overset{*}{\rightarrow} C$ in the net $\mathcal{N}$.*

Because $\mathcal{P}$ is symmetric we have the following fact, which is easy to verify.

**Proposition 8.** *If $q \in Q^F$, then $\wr 2 \cdot init^F \wr \overset{*}{\rightarrow} \wr 2 \cdot q \wr \overset{*}{\rightarrow} \wr 2 \cdot fin^F \wr$*

For any vector $\mathbf{x} \in \mathbb{N}^T$, we define $lead(\mathbf{x})$ to be the set of all leader transitions such that $\mathbf{x}[t] > 0$. The graph of the vector $\mathbf{x}$, denoted by $\mathcal{G}(\mathbf{x})$ is defined as follows: The set of vertices is the set $\{t.from : t \in lead(\mathbf{x})\} \cup \{t.to : t \in lead(\mathbf{x})\}$. The set of edges is the set $\{(t.from, t.to) : t \in lead(\mathbf{x})\}$. Further, for any two vectors $\mathbf{x}, \mathbf{y} \in \mathbb{N}^T$ and a transition $t \in T$, we say that $\mathbf{x} = \mathbf{y}[t{-}{-}]$ iff $\mathbf{x}[t] = \mathbf{y}[t] - 1$ and $\mathbf{x}[t'] = \mathbf{y}[t']$ for all $t' \neq t$.

**Definition 6.** *Let $C$ be a configuration and let $\mathbf{x} \in \mathbb{N}^T$. We say that the pair $(C, \mathbf{x})$ is compatible if $C + \mathcal{A}\mathbf{x} \geq \mathbf{0}$ and every vertex in $\mathcal{G}(\mathbf{x})$ is reachable from $lead(C)$.*

The following lemma states that *as long as there are enough followers in every state*, it is possible for the leader to come up with a firing sequence from a compatible pair.

**Lemma 9.** *Suppose $(C, \mathbf{x})$ is a compatible pair such that $C(q) \geq 2\|\mathbf{x}\|$ for every $q \in Q^F$. Then there is a configuration $D$ and a firing sequence $\xi$ such that $C \overset{\xi}{\rightarrow} D$ and $\overrightarrow{\xi} = \mathbf{x}$.*

*Proof.* (Sketch.) We prove by induction on $\|\mathbf{x}\|$. If $\mathbf{x}[t] > 0$ for some follower-only transition, then it is easy to verify that if we let $C'$ be such that $C \overset{t}{\rightarrow} C'$ and $\mathbf{x}'$ be $\mathbf{x}[t{-}{-}]$, then $(C', \mathbf{x}')$ is compatible and $C(q) \geq 2\|\mathbf{x}'\|$ for every $q \in Q^F$.

Suppose $\mathbf{x}[t] > 0$ for some leader transition. Let $p = lead(C)$. If $p$ belongs to some cycle $S = p, r_1, p_1, r_2, p_2, \ldots, p_k, r_{k+1}, p$ in the graph $\mathcal{G}(\mathbf{x})$, then we let $C \overset{r_1}{\longrightarrow} C'$ and $\mathbf{x}' = \mathbf{x}[t{-}{-}]$. It is easy to verify that $C' + \mathcal{A}\mathbf{x}' \geq \mathbf{0}$, $C'(q) \geq 2\|\mathbf{x}'\|$ for every $q \in Q^F$ and $lead(C') = p_1$. Any path $P$ in $\mathcal{G}(\mathbf{x})$ from $p$ to some vertex $s$ either goes through $p_1$ or we can use the cycle $S$ to traverse from $p_1$ to $p$ first and then use $P$ to reach $s$. This gives a path from $p_1$ to every vertex $s$ in $\mathcal{G}(\mathbf{x}')$.

If $p$ does not belong to any cycle in $\mathcal{G}(\mathbf{x})$, then using the fact that $C + \mathcal{A}\mathbf{x} \geq 0$, we can show that there is exactly one out-going edge $t$ from $p$ in $\mathcal{G}(\mathbf{x})$. We then let $C \overset{t}{\rightarrow} C'$ and $\mathbf{x}' = \mathbf{x}[t{-}{-}]$. Since any path in $\mathcal{G}(\mathbf{x})$ from $p$ has to necessarily use this edge $t$, it follows that in $\mathcal{G}(\mathbf{x}')$ there is a path from $t.to = lead(C')$ to every vertex.

**Lemma 10.** *Let $par \in \{0, 1\}$. There exists $k \in \mathbb{N}$ such that $C^k_{init} \overset{*}{\rightarrow} C^k_{fin}$ and $k \equiv par \pmod 2$ iff there exists $n \in \mathbb{N}$, $\mathbf{x} \in \mathbb{N}^T$ such that $n \equiv par \pmod 2$, $(C^n_{init}, \mathbf{x})$ is compatible and $C^n_{fin} = C^n_{init} + \mathcal{A}\mathbf{x}$.*

*Proof.* (Sketch.) The left to right implication is easy and follows from the marking equation along with induction on the number of leader transitions in the run. For the other side, we use an idea similar to Lemma 5. Let $(C^n_{init}, \mathbf{x})$ be the given compatible pair. We first use Proposition 8 to populate all the states of $Q^F$ with enough processes such that all the states of $Q^F$ except $init^F$ have an even number of processes. Then we use Lemma 9 to construct a firing sequence $\xi$ which can be fired from $C^n_{init}$ and such that $\overrightarrow{\xi} = \mathbf{x}$. By means of the marking equation, we then argue that in the resulting configuration, the leader is in the final state, $n$ followers are in the state $fin^F$ and every other follower state has an even number of followers. Once again, using Proposition 8 we can now move all the processes which are not at $fin^F$ to the final state $fin^F$.

**Lemma 11.** *Given a symmetric leader protocol, checking whether a cut-off exists can be done in NP.*

*Proof.* By Proposition 6 it suffices to find an even number $e$ and an odd number $o$ such that $C^e_{init} \xrightarrow{*} C^e_{fin}$ and $C^o_{init} \xrightarrow{*} C^o_{fin}$. Suppose we want to check that there exists $2k \in \mathbb{N}$ such that $C^{2k}_{init} \xrightarrow{*} C^{2k}_{fin}$. We first non-deterministically guess a set of leader transitions $S = \{t_1, \ldots, t_k\}$ and check that for each $t \in S$, we can reach $t.from$ and $t.to$ from $init^L$ using only the transitions in $S$.

Once we have guessed all this, we write a polynomially sized integer linear program as follows: We let $\mathbf{v}$ denote $|T|$ variables, one for each transition in $T$ and we let $n$ be another variable, with all these variables ranging over $\mathbb{N}$. We then enforce the following conditions: $C^{2n}_{fin} = C^{2n}_{init} + \mathcal{A}\mathbf{v}$ and $\mathbf{v}[t] = 0 \iff t \notin S$ and solve the resulting linear program, which we can do in non-deterministic polynomial time [26]. If there exists a solution, then we accept. Otherwise, we reject.

By Lemma 10 and by the definition of compatibility, it follows that at least one of our guesses gets accepted iff there exists $2k \in \mathbb{N}$ such that $C^{2k}_{init} \xrightarrow{*} C^{2k}_{fin}$. Similarly we can check if exists $2l+1 \in \mathbb{N}$ such that $C^{2l+1}_{init} \xrightarrow{*} C^{2l+1}_{fin}$.

By a reduction from 3-SAT, we prove that

**Lemma 12.** *The cut-off problem for symmetric leader protocols is NP-hard.*
